# Difficulty of Severe Post-liposuction Infragluteal Deformity Correction With Autologous Fat Transplantation: A Case Report

**DOI:** 10.3389/fsurg.2022.758566

**Published:** 2022-02-07

**Authors:** Mingzi Yang, Yunpeng Gu, Jingjing Sun, Qianwen Lv, Yue Qi, Ji Jin, Zhenjun Liu, Guie Ma

**Affiliations:** The 15th Department, Plastic Surgery Hospital, Chinese Academy of Medical Sciences and Peking Union Medical College, Beijing, China

**Keywords:** liposuction, fat transplantation, post-liposuction deformity, reconstruction, case report

## Abstract

Overaggressive liposuction of the infragluteal region can lead to iatrogenic infragluteal fold deformity and result in esthetic defects in the gluteal contour. We report a case of using autologous fat transplantation to correct severe post-liposuction infragluteal fold deformity. In the process of reconstruction, the patient experienced fat graft overabsorption, fat graft translocation, and gluteal ptosis aggravation. Despite multiple operations, the effect of fat transplantation was limited. In conclusion, severe post-liposuction infragluteal deformity is very difficult to correct. The infragluteal region should be preserved during liposuction to avoid deformity.

## Introduction

Contour deformity is the most common complication of liposuction, with an incidence of up to 20% (1–3). The infragluteal fold is one of the key elements that determines the gluteal contour and is recognized as an important characteristic of female beauty (4). Overaggressive liposuction of the infragluteal region can lead to iatrogenic infragluteal fold deformity and result in esthetic defects in the gluteal contour (5). Severe infragluteal fold deformity can be difficult to correct. Here, we present a case of iatrogenic deformity of the infragluteal fold region that was treated with multiple reconstructive surgeries for correction, but the result was not quite satisfactory.

## Case Description

**Figure 1:**
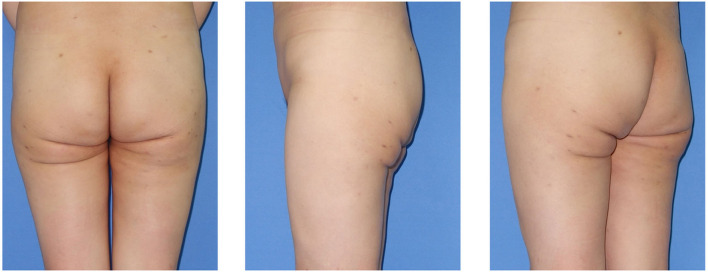
Pre-reconstruction view of the 37-year-old woman with iatrogenic infragluteal deformity. The deformity was displayed on both sides, including infragluteal region depression, multiple and asymmetric infragluteal folds, and gluteal ptosis.

A 37-year-old woman with infragluteal deformity after overaggressive gluteal and posterior thigh liposuction at a different institution came to visit and sought for correction. Physical examination showed that the deformity was displayed on both sides, including infragluteal region depression, multiple and asymmetric infragluteal folds, and gluteal ptosis (Figure 1). The patient had no history of past illness and no family history. Laboratory examinations of the patient were normal. Imaging examinations were not applicable in this case. Informed consent was obtained from the patient included in the study.

## Diagnosis

Post-liposuction infragluteal deformity.

## Treatment

Correction options were discussed with the patient. Liposuction and fat transplantation were performed while dermolipectomy and flap reconstruction were refused. Surgical techniques are described as following:

The fat transplantation was processed with the Coleman technique. The patient was marked in the standing position. The operation was performed under tumescent anesthesia. Tumescent solutions (comprising 1,000 mL normal saline, 400 mg lidocaine, and 1 mg epinephrine) were infiltrated into subcutaneous fat layer of the surgical area. The fat donor sites included the flanks, abdomen, and lateral thighs. The fat graft was harvested by suction-assisted liposuction using the syringe method. The aspirate was collected in a sterile container in which connective tissue was eliminated manually, then centrifuged at 700 g for 3 min. After discarding the upper oil layer and lower fluid, the fat graft was prepared in spiral syringes. Fat transplantation was performed in the prone position. The incisions for fat transplantation were designed in the infragluteal folds invisibility whenever possible. Fat was injected into the subcutaneous fat layer through 1.2–2.0-mm blunt-tip cannulas as the cannula was withdrawn. After the operation, the patient was instructed to avoid local pressure to the fat transplanted region for at least 3 months to prevent postoperative fat translocation.

## Outcome and Follow-Up

**Figure 2:**
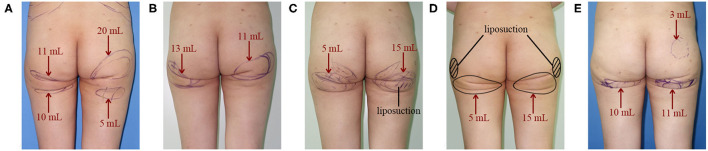
Fat transplantation region and volume of each reconstructive operation. **(A)** The 1st operation; **(B)** The 2nd operation; **(C)** The 3rd operation; **(D)** The 4th operation; **(E)** The 5th operation.

**Figure 3:**
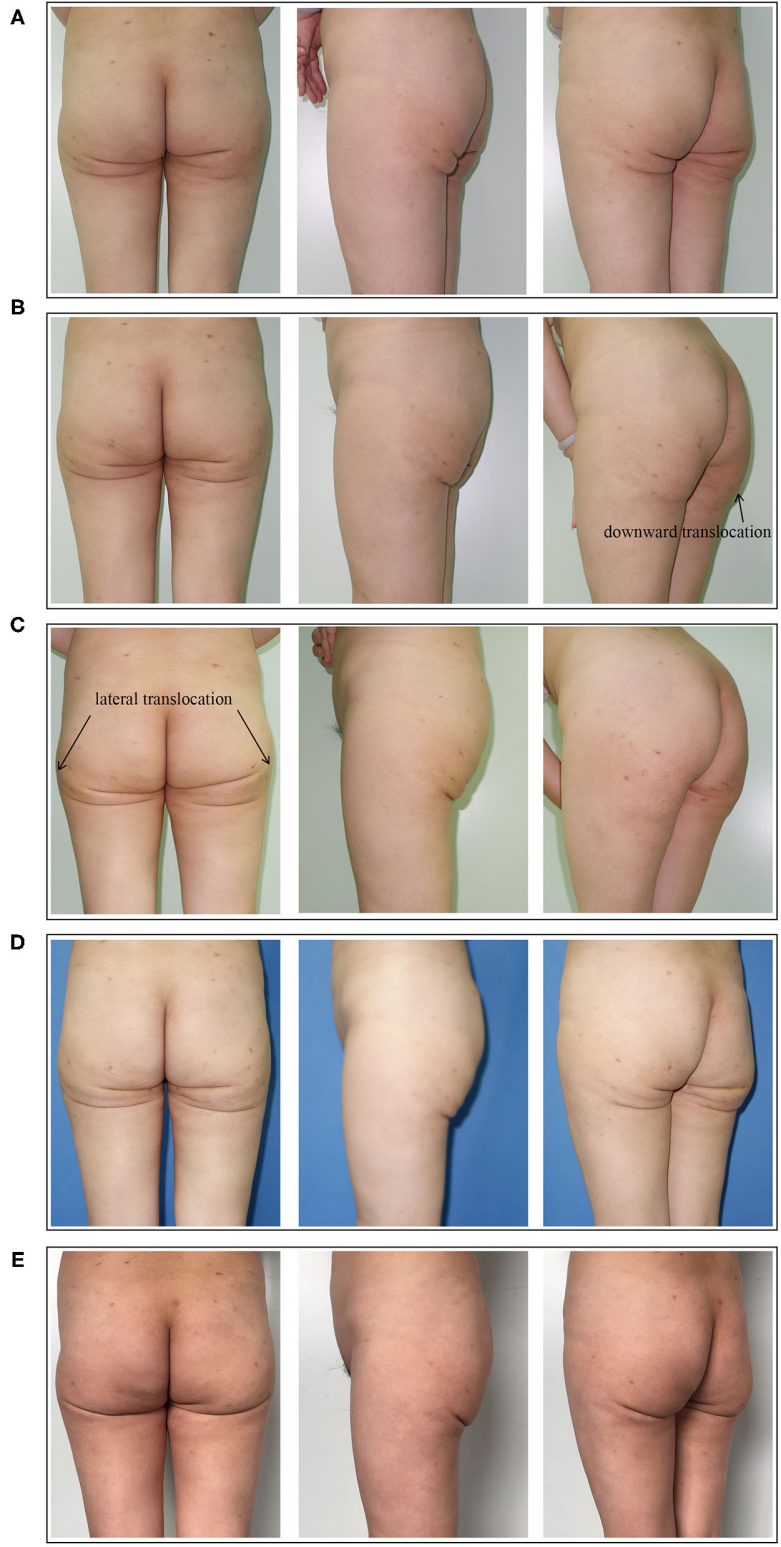
**(A)** Postoperative view of the patient 4 months after the 1st reconstructive operation. Little improvement of the deformity was seen. **(B)** Postoperative view of the patient 3 months after the 2nd reconstructive operation. The multiple infragluteal folds on the left side were slightly improved. The arrow indicates that the downward translocation of fat and the convex deformity. **(C)** Postoperative view of the patient 3 months after the 3rd reconstructive operation. The gluteal contour was improved and the convex deformity caused by fat downward translocation was corrected. The arrow indicates the lateral translocation of fat. **(D)** Postoperative view of the patient 6 months after the 4th reconstructive operation. The multiple infragluteal folds were further improved to a small extent. **(E)** Postoperative view of the patient 8 months after the 5th reconstructive operation. The multiple and asymmetric folds were significantly improved. The infragluteal crease lines were extended and gluteal ptosis was aggravated in appearance.

A total of five reconstructive operations were performed. The fat transplantation region and volume of each operation are shown in Figure 2. Four months after the 1st operation, the fat graft was almost entirely absorbed, and little improvement of the deformity was seen (Figure 3A). Three months after the 2nd operation, the multiple infragluteal folds on the left side were slightly improved. However, the fat graft translocated downward, which led to a convex deformity (Figure 3B). Three months after the 3rd operation, the lateral view of the gluteal contour was improved, and the convex deformity caused by the 2nd operation was corrected. Nevertheless, the fat graft translocated laterally and distributed in the hip area (Figure 3C). Six months after the 4th operation, the multiple infragluteal folds were further improved to a small extent (Figure 3D). Finally, 8 months after the 5th operation, although the multiple and asymmetric folds were significantly improved, the infragluteal crease lines were extended, and severe gluteal ptosis appeared (Figure 3E).

**Figure 4:**
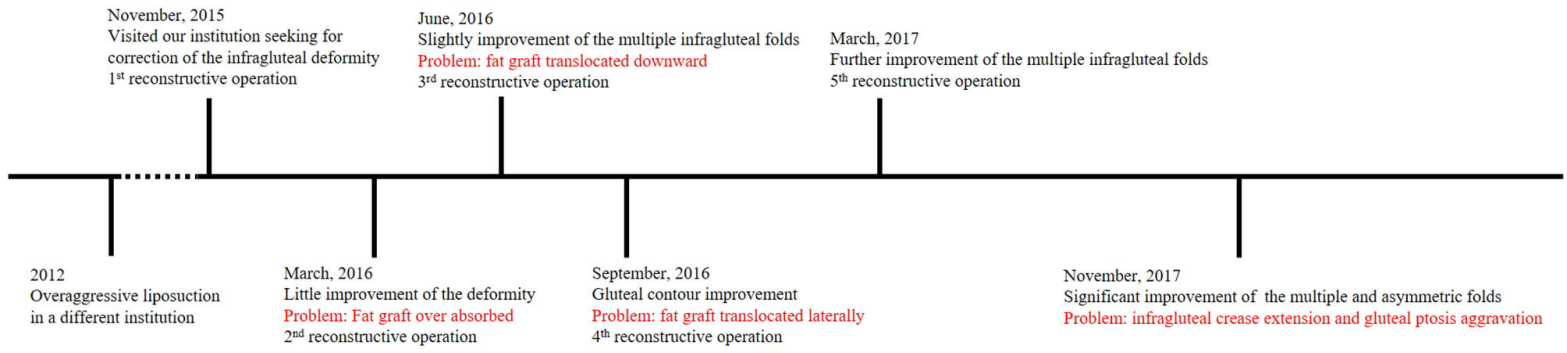
An outline of the reconstructive process.

In summary, after 5 reconstructive surgeries, the depression deformity and multiple, asymmetric fold deformities were partially corrected but not quite satisfactorily, and gluteal ptosis was aggravated in appearance. In the process of reconstruction, the patient experienced fat graft overabsorption, fat graft translocation, infragluteal crease extension, and gluteal ptosis aggravation. An outline of the reconstructive process is shown in Figure 4.

## Discussion

According to previous reports, the correction of post-liposuction infragluteal fold deformity mainly depends on flap reconstruction, liposhifting and autologous fat transplantation (5–8). Flap reconstruction is efficient in severe cases of infragluteal fold deformity (5), but the low patient acceptance of the subsequent scars limits its application. Liposhifting is a technique that frees subcutaneous fat by stab incisions and cannulas and shifts the surrounding fat to correct depression deformities without liposuction or fat injection (6). However, this procedure may be insufficient for correcting large deformities with severe adherence. In regard to the case we presented, fat transplantation was considered the optimal option given refusal of flap reconstruction. However, the result was still not quite satisfactory after multiple operations.

As far as we are concerned, the difficulty in correcting post-liposuction infragluteal deformities may be mainly due to the subcutaneous scar. Anatomically, the infragluteal fold region is described as an adherence zone in which the superficial fascial tissue fuses and firmly adheres to the deep gluteal fascia in the absence of a deep layer fat (9, 10). This is critically important in the formation of the infragluteal fold and caudal gluteal border (10). In the case of overaggressive liposuction, the superficial fascia is almost compensated by scar tissue, and the dense fibrous attachment to the underlying deep fascia is broken (11). Fat grafts are difficult to fill and survive in scar tissue; thus, volume maintenance is difficult to predict. This would result in the inefficient of fat transplantation. Moreover, because of the loss of attachment between the scar and deep fascia, the transplanted fat barely contacted the adjacent healthy tissue. Inevitably, the fat graft is prone to translocation, for instance, downward and lateral translocation presented in our patient, even if local pressure to the transplantation region is avoided as much as possible after the operation. Therefore, the effect of fat transplantation in the correction of severe post-liposuction infragluteal deformity is limited.

Except for the multiple infragluteal folds, the correction of gluteal ptosis after overaggressive liposuction could be more difficult. The post-liposuction gluteal ptosis may be associated with the destruction of conjunctive fibrous tissue that sustains the buttocks and skin laxity subsequent to soft tissue volume reduction (11). Generally, the evaluation of gluteal ptosis is determined by the length of the infragluteal crease and the amount of sagging tissue passing over the infragluteal fold (4, 12, 13). With respect to our patient, after the 5th fat transplantation, although the soft tissue volume of the infragluteal region was supplemented, without intact sustaining structures, the increased volume and redundant skin sagged, and the infragluteal crease was extended. As a result, the gluteal ptosis was aggravated. Therefore, we recognize that fat transplantation is problematic in improving severe post-liposuction gluteal ptosis, and the infragluteal region should be preserved as possible during liposuction to avoid deformity.

## Conclusion

Post-liposuction infragluteal deformity is very difficult to correct. It is important to recognize that subcutaneous scar formation, conjunctive fibrous tissue destruction and soft tissue volume reduction after liposuction may result in infragluteal deformity, and the effect of fat transplantation is limited when dealing with severe deformities. In our opinion, the infragluteal region should be preserved as possible during liposuction to avoid deformity.

## Patient Perspective

The whole repair process took a long time. Although the infragluteal deformity was improved after multiple reconstructive surgeries, the gluteal contour did not achieve very natural.

## Data Availability Statement

The raw data supporting the conclusions of this article will be made available by the authors, without undue reservation.

## Ethics Statement

Written informed consent was obtained from the individual(s) for the publication of any potentially identifiable images or data included in this article.

## Author Contributions

GM, MY, and YG designed this study. MY and YG completed the article. JS, QL, YQ, JJ, and ZL helped to collect data for this study. GM revised the article. All authors contributed to the article and approved the submitted version.

## Funding

This study was supported by the Medical and Health Science Innovation Project of the Chinese Academy of Medical Sciences (Medical Big Data Information Collection and Analysis Evaluation, Fund No. 2016-12M-2-004).

## Conflict of Interest

The authors declare that the research was conducted in the absence of any commercial or financial relationships that could be construed as a potential conflict of interest.

## Publisher's Note

All claims expressed in this article are solely those of the authors and do not necessarily represent those of their affiliated organizations, or those of the publisher, the editors and the reviewers. Any product that may be evaluated in this article, or claim that may be made by its manufacturer, is not guaranteed or endorsed by the publisher.
